# Design and Synthesis of New *N*-(5-Trifluoromethyl)-1*H*-1,2,4-triazol-3-yl Benzenesulfonamides as Possible Antimalarial Prototypes

**DOI:** 10.3390/molecules16098083

**Published:** 2011-09-20

**Authors:** Nubia Boechat, Luiz C.S. Pinheiro, Osvaldo A. Santos-Filho, Isabor C. Silva

**Affiliations:** Departamento de Sintese Organica, Instituto de Tecnologia em Farmacos, Fundacao Oswaldo Cruz, Manguinhos, CEP 21041-250, Rio de Janeiro, RJ, Brazil

**Keywords:** malaria, sulfonamide, trifluoromethyl, dihydropteroate synthase, DHPS

## Abstract

A rational approach was used to synthesize a new set of 15 1*H*-1,2,4-triazol-3-yl benzenesulfonamide derivatives with the aim of developing new antimalarial lead compounds. These derivatives were prepared in yields between 50% and 62%, and their structures were elucidated using IR, ^1^H-, ^13^C-, ^19^F-NMR, MS and elemental analysis. A docking study based on sulfonamides previously used against malaria identified trifluoromethyl-substituted derivatives to be the best lead compounds for new antimalarial drug development.

## 1. Introduction

Malaria is a deadly mosquito-borne disease that afflicts as many as half a billion people; about 250 million malaria cases and nearly one million associated deaths have been reported in 106 countries across Africa, Asia and Latin America [1]. Drug resistance, documented in *Plasmodium falciparum*, *P. malariae* and *P. vivax* malaria species, has been one of the main obstacles in the fight against malaria. Increasing resistance to antimalarial drugs has a number of implications for the eradication of malaria. Inappropriate use of antimalarial drugs and the use of monotherapies or substandard and counterfeit medicines are suggested to be the main contributors to widespread resistance in malaria parasites [2].

Chemotherapeutic treatment of malaria can involve a number of compounds: quinine, chloroquine, mefloquine, primaquine, amodiaquine, proguanil, pyrimethamine, sulfadoxine, artemisinin, halofantrine, pyronaridine, piperaquine, and artemisinin derivatives, such as artemether, artesunate and dihydroartemisinin. However, in the absence of an effective vaccine, and given the decreased efficiency of classical medications against chloroquine-resistant strains, new antimalarial drugs that are efficient, safe and synthetically economical are needed [3,4].

Organofluorine compounds have been broadly explored in medicinal chemistry and play a continuing role as lead compounds for therapeutic applications. The introduction of a fluorine atom has been an important strategy in the modification of chemical reactivity and physical and biological properties of organic compounds. This strategy can critically influence pharmacodynamic and pharmacokinetic properties, reinforce drug-receptor interactions, aid absorption and translocation across lipid bilayers and induce conformational changes to block metabolism [5,6,7,8,9,10,11].

One of the most widespread fluorine-containing functional groups in bioactive molecules is the trifluoromethyl moiety [12,13], a highly electronegative substituent that can exert significant electronic influence on neighboring groups [9]. The trifluoromethyl group can also cause dramatic steric change, as its van der Waals volume is comparable to that of an ethyl [12] or isopropyl [6] group, although with a significantly different shape. It is among the most lipophilic groups known and is therefore useful in improving the targeting of molecules to active sites [5,6]. Mefloquine, one of the most important antimalarial agents, contains two trifluoromethyl groups [2]. Recent literature on compounds that are active against malaria reports a large number of new molecules containing trifluoromethyl groups [14,15,16].

Triazoles are important members of the azole class of antiparasitic drugs. They have been used to treat cutaneous leishmaniasis and/or Chagas’ disease with variable rates of success [17,18], and they possess antimalarial activity [19,20,21,22,23].

Sulfonamides are widely used in medicinal chemistry because of their low cost, low toxicity and excellent biological activity. For example, sulfadoxine, sulfadiazine, and sulfalene are effective malaria drugs that possess sulfonamide groups attached to a heterocyclic ring. Krungkrai and coworkers reported a library of aromatic/heteroaromatic sulfonamides with diverse scaffolds and assayed these compounds for the inhibition of carbonic anhydrase from *Plasmodium falciparum* (pfCA) [24,25]. The literature describes several other sulfonamides with antimalarial activity [26,27,28,29,30,31,32]. Some of these studies involve the inhibition of folate metabolic enzymes that are crucial for the growth of the malaria parasite. Some of these enzymes are absent in humans and are therefore potential targets for malarial chemotherapy. However, parasitic resistance to currently used drugs has spread worldwide, and the scientific community is researching alternatives.

A promising approach is the development of fixed-dose combinations of antimalarial agents, for example, a pyrimethamine-sulfadoxine combination (Fansidar^®^), which has been widely used to treat *P. falciparum* in Africa, where these parasites are resistant to chloroquine [33]. Pyrimethamine inhibits the enzyme dihydrofolate reductase (DHFR) whereas sulfadoxine inhibits dihydropteroate synthase (DHPS) [34,35,36].

**Scheme 1:**
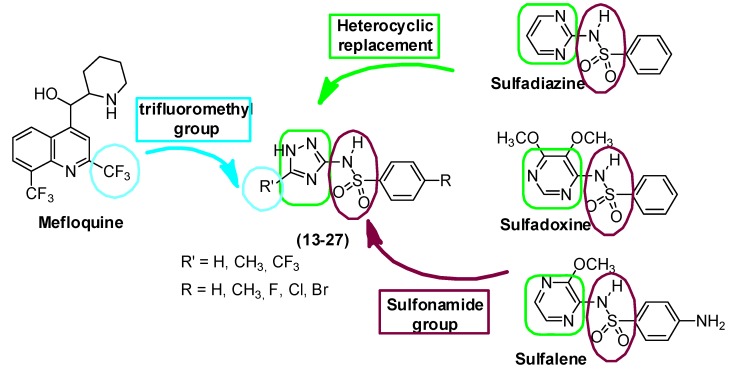
Rational approach to design the 1*H*-1,2,4-triazol-3-yl benzenesulfonamide derivatives **13–27**.

The literature describes the synthesis and biological activity of some 1*H*-1,2,4-triazol-3-yl benzenesulfonamides [37,38,39]. We sought to develop a potent and economical series of synthetic 1*H*-1,2,4-triazol-3-trifluoromethyl benzenesulfonamides as antimalarial agents. By using traditional medicinal chemistry principles, we designed and synthesized a set of derivatives **13–27** as potential antimalarial compounds (Scheme 1). Using bioisosteric principles, the heterocyclic ring of the sulfonamides drugs (sulfadoxine, sulfadiazine, and sulfalene) was replaced by a triazole ring in the designed compounds. Different substituents were incorporated to investigate the importance of the group at position 5 on the triazole ring. The CF_3_ present in mefloquine was prioritized. We also varied the group at position 4' of the aromatic ring to investigate its influence. The sulfonamide derivatives were synthesized, nine new **13–17**, **19**, **20**, **24**, **25**, and evaluated by molecular docking. Here, we report the synthesis and modeling of 1*H*-1,2,4-triazol-3-yl benzenesulfonamides that potentially inhibit *P. falciparum* DHPS.

### 1.1. Chemistry

**Scheme 2:**
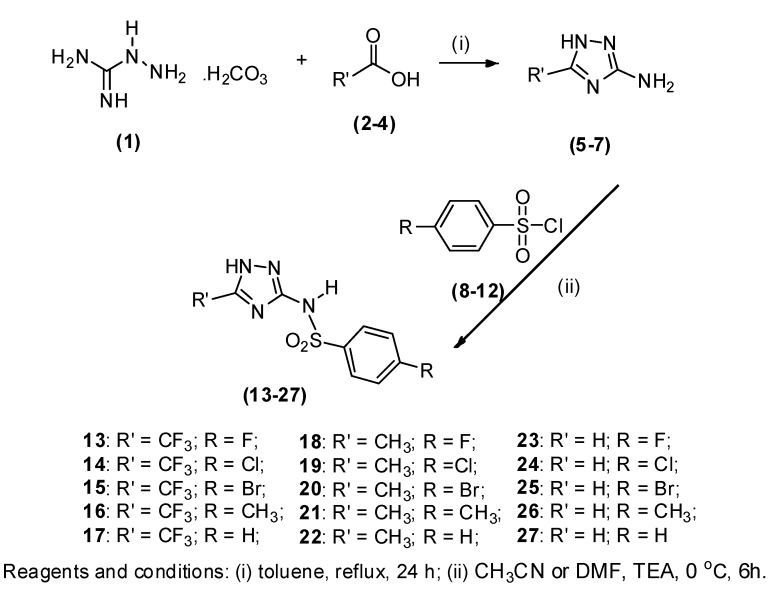
Synthetic approach used to obtain the 1*H*-1,2,4-triazol-3-yl benzenesulfonamide derivatives **13–27**.

The synthetic route for preparing the 1*H*-1,2,4-triazol-3-yl benzenesulfonamide derivatives **13–27** is illustrated in Scheme 2. The 3-amine-1*H*-1,2,4-triazoles **5–7** were prepared in excellent yield (91%–99%) using aminoguanidine bicarbonate (**1**) to undergo condensation followed by cyclization with the appropriate carboxylic acid **2–4**.

To produce the target compounds, an equimolar mixture of 3-amino-1*H*-1,2,4-triazoles **5–7** and the appropriate sulfonyl chloride derivative **8–12** was stirred in the presence of acetonitrile or DMF at room temperature to produce the 1*H*-1,2,4-triazol-3-yl benzenesulfonamides **13–27**. Products were isolated in moderate yields (50%–62%) and characterized spectroscopically using IR, ^1^H-, ^13^C-, ^19^F-NMR, MS and elemental analysis.

### 1.2. Molecular Modeling and Docking Simulation

Each target molecule was modeled *in silico*, and energy minimization was performed over 1,000 steps using the steepest descent method, Gasteiger-Hückel charges, a dielectric constant of 80, and the Tripos force field. The structures were further optimized using the conjugated gradient method.

A model of the active site of *P. falciparum* DHPS was constructed *in silico* from the crystal structure of *Escherichia coli* DHPS (PDB entry: 1AJ0) [40], which includes a sulfonamide in its active site. Ligand-enzyme docking simulations were performed with the molecular docking algorithm MolDock [41]. This software uses a heuristic search algorithm (termed “guided differential evolution”) that combines differential evolution and a cavity-prediction algorithm. The docking scoring function is an extension of the piecewise linear potential (PLP) [41]. After the ligands and the protein coordinates were imported, all structural parameters, including bond type, hybridization, explicit hydrogens, charges, and flexible torsions, were assigned using the automatic preparation function in Molegro Virtual Docker software. For each compound, 100 docking runs were performed with an initial population of 150 individuals. After docking, each compound was energy minimized into the active site of the enzyme.

## 2. Results and Discussion

The preparation of 3-amino-1*H*-1,2,4-triazoles **5–7** was achieved through condensation followed by cyclization of aminoguanidine bicarbonate (**1**) and the appropriate carboxylic acid **2–4**. The triazoles **5–7** were obtained as white powders in excellent yields ranging from 91% to 99%. The 3-amino-1*H*-1,2,4-triazoles **5–7** were used directly in the next step without purification. The chemical structures of **5–7** were confirmed by analyzing the crude product by ^1^H-, ^13^C- and ^19^F-NMR, as well IR. An IR analysis showed an absorption band at 3456–3208 cm^−1^, attributed to the stretch vibrations of the NH bond of the amino group.

The nucleophilic substitution reactions between 3-amino-1*H*-1,2,4-triazoles **5–7** and sulfonyl chloride derivatives **8–12** were performed under magnetic stirring at room temperature in acetonitrile or DMF, for derivatives **5–6** and **7**, respectively, to prepare 1*H*-1,2,4-triazol-3-yl benzenesulfonamide derivatives **13–27** in moderate yields (50%-62%). Derivatives **13–27** were identified using FT-IR; the spectra lacked the previous diagnostic stretching vibration of the NH bond of the amino group and included bands from the sulfonamide at 1,396–1,340 and 1,188–1,141 cm^−1^. In the ^1^H-NMR spectra, the signals of the respective protons of the synthesized compounds were verified on the basis of their chemical shifts, multiplicities and coupling constants. The ^1^H-NMR spectra showed double doublet or multiplet signals corresponding to the aromatic hydrogens. The triazole protons were observed as a singlet at 7.48–8.32 ppm for compounds **23–27**, whereas compounds **18–22** showed another singlet at 1.96–2.00 ppm for the CH_3_ group. Compounds **13–17**, which contain trifluoromethyl groups, were confirmed mainly by characteristic ^19^F-NMR signals at −64.72 to −68.16 ppm. The ^13^C-NMR spectra showed characteristic quartet signals at 118.42–118.45 ppm with *J* = 269 Hz, for the CF_3_ group, and a quartet signal at 152.55–152.44 ppm with *J* = 39 Hz, for C-5. The *p*-F substituted compounds **13**, **18**, and **23** showed C-F as a doublet signal at 165.66–166.21 ppm with *J* = 255 Hz, for the C4', a doublet signal at 117.34–117.77 ppm with *J* = 23 Hz for C3' and C5', and a doublet signal at 130.83–131.38 ppm with *J* = 10 Hz, for C2' and C6'.

### 2.1. Molecular Modeling and Docking

*P. falciparum* DHPS is a bifunctional enzyme that includes dihydro-6-hydroxymethylpterin pyrophosphokinase (PPPK) at the N-terminus of DHPS. No crystal structure is yet available for this malarial enzyme, but its primary structure is available from proteome databases. We therefore used GenBank to acquire and study the nucleotide sequence of *P. falciparum* DHPS (GenBank entry: Q27865) [42]. A preliminary comparative model was constructed for the entire *P. falciparum* DHPS protein. However, the overall score evaluation for this model was unsatisfactory; therefore, we performed energy minimization with the AMBER force field [43]. The resulting optimized structure still displayed a number of structural problems. This low resolution led us to discontinue our planned structure-based design experiments using the model.

Since the active site residues of enzymes are well conserved through molecular evolution, we decided to search for DHPS crystal structures from other organisms that could be used as templates for modeling the active site of the *P. falciparum* enzyme. The malarial sequence (Q27865) was submitted to the Basic Local Alignment Search Tool (BLAST) [44]. The crystal structures of DHPS from several sources showed high local score similarity with the malarial enzyme, justifying their use in modeling our active site. Because the *Escherichia coli* DHPS (PDB entry: 1AJ0) [40] was crystallized with a sulfonamide bound to its active site, we used that structure as our main template.

**Figure 1:**
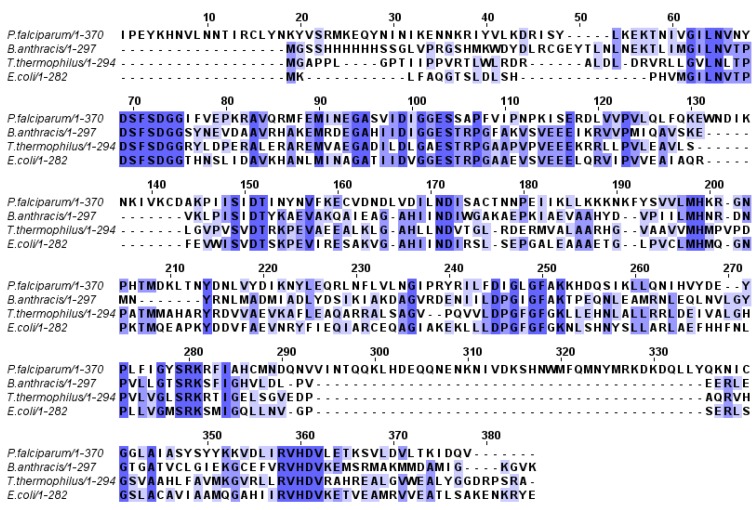
Multiple sequence alignment of the malarial enzyme and the top-3 similar sequences.

Figure 1 shows a multiple sequence alignment of the top three matches for malarial DHPS. By analyzing both the sequence alignment and the crystal structure of the bacterial enzyme (PDB entry: 1AJ0), it was possible to infer the active site of the malarial enzyme. Table 1 shows the active site residues from the bacterial enzyme and the corresponding malarial residues. Notably, just three of the 15 active-site residues from *E. coli* enzyme differ from the *P. falciparum* enzyme active site. We modified the appropriate residues for the *E. coli* DHPS and performed an energy minimization on each mutated residue and their neighbor residues. A search for the best dihedral angles for the residues involved ensured that structural clashes had been removed from the model.

**Table 1:** Active site residues of both *E. coli and P. falciparum* DHPS’s.

Active site residues of DHPS		
N°.	*E. coli* (PDB entry: 1AJ0)	*P. falciparum* model from site-direct mutagenesis from 1AJ0
22	N	N
**62**	**T**	**S**
**63**	**R**	**A**
96	D	D
115	N	N
117	I	I
185	D	D
190	F	F
**215**	**L**	**F**
217	G	G
219	S	S
220	R	R
221	K	K
255	R	R
257	H	H

**Figure 2:**
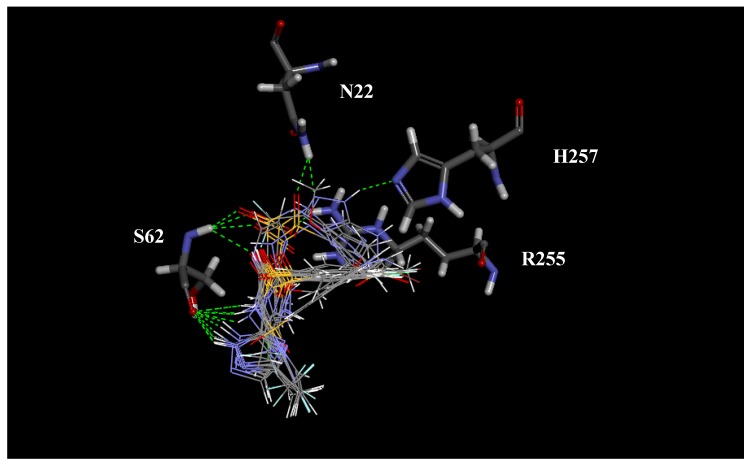
Intermolecular hydrogen bonds between *P. falciparum* DHPS and the synthesized ligands, sulfadoxine, sulfadiazine, and sulfalene.

**Figure 3:**
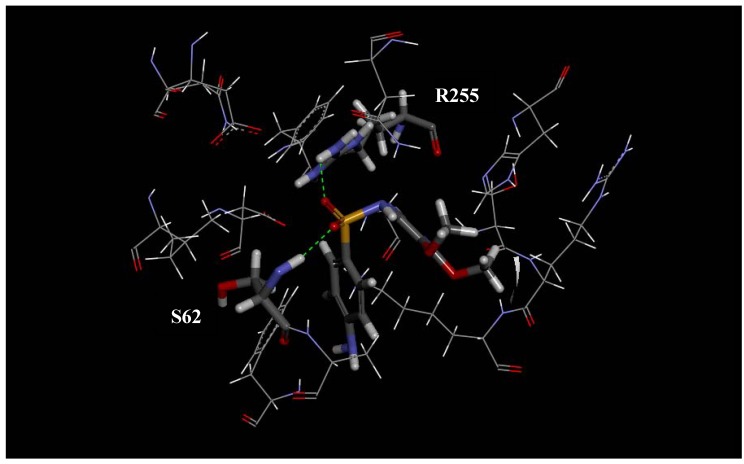
Docking of sufadoxine into *P. falciparum* DHPS.

**Figure 4:**
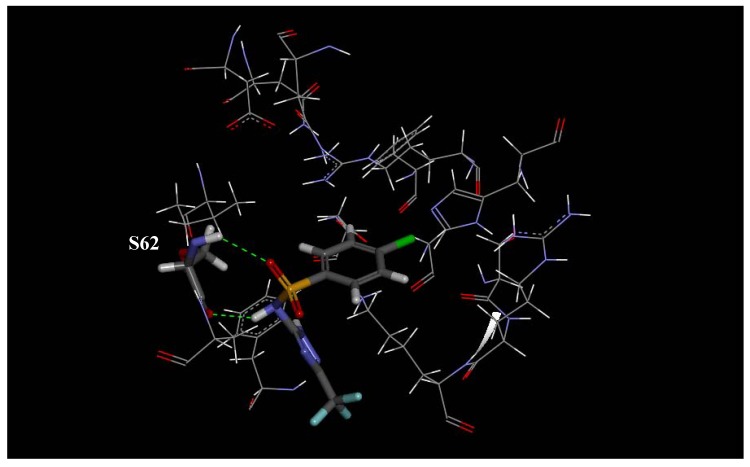
Docking of **14** into *P. falciparum* DHPS.

The docking score of the interactions between the active site of *Pf*DHPS and each synthesized molecule, as well as sulfadoxine, sulfadiazine and sulfalene, are shown in Table 2. Figure 2, Figure 3 and Figure 4 show specific receptor-ligand interactions in detail. There are a number of strong interactions between the ligands and residues N22, S62, H257, and R255 that stabilize the intermolecular geometry. Another interesting result from the docking calculation is the alignment of the triazole rings of all compounds.

**Table 2:** Docking score.

**Ligand**	Docking Score (kcal/mol)
**14**	−93.42
**15**	−91.03
**16**	−89.00
**13**	−88.89
**20**	−88.65
**18**	−87.73
**19**	−87.24
**17**	−85.98
**25**	−83.87
**23**	−83.85
**21**	−83.48
sulfalene	−78.78
sulfadoxine	−78.01
**22**	−76.55
**26**	−76.17
**24**	−76.15
**27**	−71.79
sulfadiazine	−70.03

According to the docking score results (Table 2), compounds **13–16** with CF_3_ groups on position 5 of the triazole ring, but not compound **17**, showed stronger interaction with the enzyme. The non-substituted triazole analogs **23–27** exhibited the weakest intermolecular interactions with the enzyme.

All of the halogenated compounds **13–15**, **18–20**, **23–25**, except compound **24**, presented better docking scores than did sulfalene and sulfadoxine (Table 2).

## 3. Experimental

### 3.1. General

The ^1^H-, ^13^C- and ^19^F-Nuclear Magnetic Resonance (NMR) spectra were obtained at 400.00, 100.00 and 376.00 MHz, respectively, on a Bruker Advance instrument, equipped with a 5 mm probe, or at 300.00, 75.0 and 188.00 MHz, respectively, on a Varian model Unity Plus spectrometer, using tetramethylsilane as internal standard. The chemical shifts (δ) reported in ppm and the coupling constants (*J*) in Hertz. Fourier transform infrared (FT-IR) absorption spectra were recorded on a Shimadzu mode IR Prestige-21 spectrophotometer by reflectance in KBr. GC/MS employing a model 6890N gas chromatograph (Agilent, Palo Alto, CA, USA), equipped with a 7683B auto sampler and coupled to a model MS 5973N single quadrupole mass spectrometer (Agilent). The GC is equipped with a HP-5MS capillary column 30 m length, 0.25 mm diameter, 0.25 μm film thickness, in the temperature program: 50 °C then increased to 300 °C at a rate oh 10 °C /min and kept for 10 min. the helium flow rate was 0.5 mL/min. Melting points (m.p.) were determined with a Büchi model B-545 apparatus. TLC was carried out using silica gel F-254 Glass Plate (20 × 20 cm). All other reagents and solvents used were analytical grade.

### 3.2. General Procedure for Preparing 3-Amine-1H-1,2,4-triazoles ***5–7***

A mixture of aminoguanidine bicarbonate (**1**) and the appropriate organic acid **2–4** (1 equivalent) was stirred at room temperature to complete release of CO_2_ and then toluene (150 mL) was added. The reaction mixture was heated to reflux with a Dean-Stark apparatus under magnetic stirring for 20 h. The resulting white solid was then cooled to RT, filtered, washed with toluene, and dried. Compounds **5–7** were used without purification.

*3-Amine-5-(trifluoromethyl)-1H-1,2,4-triazole* (**5**). Yield: 91%. m.p. 200 °C. IR (KBr, cm^−1^): 3,441; 3,355 (ν NH); 1,654 (ν C=N); 1,580; 1,541 (δ NH); 1,347; 1,141; 780 (ν CF_3_). ^1^H-NMR (300 MHz, DMSO-d_6_, δ in ppm): 12.24 (s; 1H; NH); 6.0 (s; 2H; NH_2_). ^13^C-NMR (75 MHz, DMSO-d_6_, δ in ppm): 148.7 (C-3); 140.8 (q; *J* = 36 Hz; C-5); 110.5 (q; *J* = 267 Hz; CF_3_). ^19^F-NMR (188 MHz, DMSO-d6, δ in ppm): −64.79 (s, 3F, CF_3_). MS (70 eV) *m/z* (%): 152 (100); 133 (25); 104 (24); 69 (59).

*3-Amine-5-methyl-1H-1,2,4-triazole* (**6**). Yield: 99%. m.p. 265 °C. IR (KBr, cm^−1^): 3,456; 3,208 (ν NH); 1,672 (ν C=N); 1,554 (δ NH); 1,345 (ν CH_3_); 1,070–1,017 (ν C–N). ^1^H-NMR (300 MHz, DMSO-d_6_, δ in ppm): 8.7–8.0 (s; 2H; NH_2_); 6.3–5.8 (s; 1H; NH); 2.13 (s; 3H; CH_3_). ^13^C-NMR (75 MHz, DMSO-d_6_, δ in ppm): 159.9 (C-3); 154.9 (C-5); 13.2 (C-6). MS (70 eV) *m/z* (%): 98 (100); 57 (86).

*3-Amine-1H-1,2,4-triazole* (**7**). Yield: 93%. m.p. 142 °C. IR (KBr, cm^−1^): 3,413–3,332 (ν NH); 2,842 (ν C–H); 1,644 (ν C=N); 1,594 e 1,538 (δ N–H); 1,373–1,213 (δ C-N). ^1^H-NMR (300 MHz, DMSO-d_6_, δ in ppm): 12.1 (s; 1H; NH); 7.5 (s; 1H; H5); 6.3–5.4 (s; 2H; NH_2_). ^13^C-NMR (75 MHz, DMSO-d_6_, δ in ppm): 158.0 (C-3); 147.7 (C-5). MS (70 eV) *m/z* (%):84 (100); 57 (45).

### 3.3. General Procedure for Preparing 1H-1,2,4-Triazol-3-yl benzenesulfonamides ***13–27***

The appropriate sulfonyl chloride **8–12** (1.2 mmol) was treated with the respective 3-amine-1*H*-1,2,4-triazole (1 mmol) in acetonitrile (for **5**, **6**) or DMF(for **7**) at 0 °C. The mixture was reacted at room temperature for 6 h and then poured into ice-water. The precipitate was collected by filtration, washed with iced-acetonitrile, dried and recrystallized in ethanol.

*4-Fluoro-N-(5-trifluoromethyl)-1H-1,2,4-triazol-3-yl benzenesulfonamide* (**13**). Yield: 52%. m.p. 175–176 °C. IR (KBr, cm^−1^): 3,448; 3,176; 1,649; 1,384; 1,201; 1,141; 709. ^1^H-NMR (400 MHz, DMSO-d_6_, δ in ppm): 8.21–7.60 (m, 5H, H–Ar); 7.95 (s, NH). ^13^C-NMR (100 MHz, DMSO-d_6_, δ in ppm): 166.21 (d, *J* = 255 Hz, C4'); 157.72; 152.44 (q, *J* = 39 Hz, C-5); 131.62; 131.38 (d, *J* = 10 Hz, C2', C6'); 118.45 (q, *J* = 270 Hz, CF_3_); 117.77 (d, *J* = 23 Hz, C3', C5'). ^19^F-NMR (376 MHz, DMSO-d_6_, δ in ppm): −66.45 (s, 3F, CF_3_); −100.41 (s, 1F). MS *m/z* (%): 310; 159; 95 (100). Anal. Calc. for: C_9_H_6_F_4_N_4_O_2_S (310.01) C, 34.84; H, 1.95; N, 18.06; found: C, 35.46; H, 1.21; N, 17.93.

*4-Chloro-N-(5-trifluoromethyl)-1H-1,2,4-triazol-3-yl benzenesulfonamide* (**14**). Yield: 51%. m.p. 203–205 °C. IR (KBr, cm^−1^): 3,475; 3,197; 1,643; 1,396; 1,149; 759. ^1^H-NMR (400 MHz, acetone-d_6_, δ in ppm): 8.14 (d, *J* = 8 Hz, 2H, H3', H5'); 7.81 (d, *J* = 8 Hz, 2H, H2', H6'); 7.29 (s, NH). ^13^C-NMR (100 MHz, DMSO-d_6_, δ in ppm): 157.78; 152.55 (q, *J* = 39 Hz, C-5); 141.27; 134.11; 130.50; 129.76; 118.42 (q, *J* = 269 Hz, CF_3_). ^19^F-NMR (376 MHz, DMSO-d_6_, δ in ppm): −68.16 (s, 3F, CF_3_). MS *m/z* (%): 326; 175 (100); 111. Anal. Calc. for: C_9_H_6_ClF_3_N_4_O_2_S (325.98) C, 33.09; H, 1.85; N, 17.15; found: C, 33.20; H, 1.81; N, 16.94.

*4-Bromo-N-(5-trifluoromethyl)-1H-1,2,4-triazol-3-yl benzenesulfonamide* (**15**). Yield: 50%. m.p. 194–196 °C. IR (KBr, cm^−1^): 3,473; 3,170; 1,643; 1,394; 1,186. ^1^H-NMR (400 MHz, DMSO-d_6_, δ in ppm): 7.91 (d, *J* = 8.8 Hz, 2H, H3', H5'); 7.83 (d, *J* = 8.8 Hz, 2H, H2', H6'); 7.52 (s, NH). ^13^C-NMR (100 MHz, DMSO-d_6_, δ in ppm): 157.77; 152.53 (q, *J* = 39 Hz, C-5); 134.53; 133.47; 132.87; 128.28; 118.42 (q, *J* = 269 Hz, CF_3_). ^19^F-NMR (376 MHz, DMSO-d_6_, δ in ppm): −64.72 (s, 3F, CF_3_). MS *m/z* (%): 370; 219 (100); 155. Anal. Calc. for: C_9_H_6_BrF_3_N_4_O_2_S (369.93) C, 29.13; H, 1.63; N, 15.10; found: C, 31.57; H, 1.39; N, 14.65.

*4-Methyl-N-(5-trifluoromethyl)-1H-1,2,4-triazol-3-yl benzenesulfonamide* (**16**). Yield: 53%. m.p. 170–172 °C. IR (KBr, cm^−1^): 3,448; 3,091; 1,591; 1,371; 1,174;700. ^1^H-NMR (400 MHz, DMSO-d_6_, δ in ppm): 7.48 (d, *J* = 10 Hz, 2H, H3', H5'); 7.13 (d, *J* = 10 Hz, 2H, H2', H6'); 2,28 (s, 3H, CH_3_). ^13^C-NMR (100 MHz, DMSO-d_6_, δ in ppm): 157.11; 152.76 (q, *J* = 39 Hz, C-5); 140.78; 135.85; 130.91; 129.21; 118.63 (q, *J* = 269 Hz, C-9); 21.37. ^19^F-NMR (376 MHz, DMSO-d_6_, δ in ppm): −66.39 (s, 3F, CF_3_). MS *m/z* (%): 306; 155; 91 (100). Anal. Calc. for: C_10_H_9_F_3_N_4_O_2_S (306.03) C, 39.22; H, 2.96; N, 18.29; found: C, 39.71; H, 2.88; N, 18.71.

*N-(5-Trifluoromethyl)-1H-1,2,4-triazol-3-yl benzenesulfonamide* (**17**). Yield: 53%. m.p. 133–135 °C. IR (KBr, cm^−1^): 3,298; 2,775; 1,693; 1,591; 1,357; 1,176; 732. ^1^H-NMR (400 MHz, DMSO-d_6_, δ in ppm): 8.08–7.73 (m, 5H, Ar); 7.92 (s, NH). ^13^C-NMR (100 MHz, DMSO-d_6_, δ in ppm): 157.79; 152.41 (q, *J* = 39 Hz, C-5); 136.02; 135.43; 130.26; 127.78; 118.45 (q, *J* = 269 Hz, C-9). ^19^F-NMR (376 MHz, DMSO-d_6_, δ in ppm): −66.45 (s, 3F, CF_3_). MS *m/z* (%): 292; 141; 77 (100). Anal. Calc. for: C_9_H_7_F_3_N_4_O_2_S (292.02) C, 36.99; H, 2.41; N, 19.17; found: C, 37.05; H, 2.25; N, 18.85.

*4-Fluoro-N-(5-methyl-1H-1,2,4-triazol-3-yl) benzenesulfonamide* (**18**). Yield: 57%. m.p. 281–283 °C. IR (KBr, cm^−1^): 3,107; 1,593; 1,363; 1,188. ^1^H-NMR (400 MHz, DMSO-d_6_, δ in ppm): 8.05–7.11 (m, 4H, H–Ar); 7.24 (s, NH); 1.96 (s, 3H, CH_3_). ^13^C-NMR (100 MHz, DMSO-d_6_, δ in ppm): 165.66 (d, *J* = 253 Hz, C4'); 161.55; 157.24; 132.74; 130.83 (d, *J* = 10 Hz, C2', C6'); 117.34 (d, *J* = 24 Hz, C3', C5'); 13.90. ^19^F-NMR (376 MHz, DMSO-d_6_, δ in ppm): −101.57 (s, 1F). MS *m/z* (%): 256 (100); 95; 75. Anal. Calc. for: C_9_H_9_FN_4_O_2_S (256.04) C, 42.18; H, 3.54; N, 21.86; found: C, 30.46; H, 4.54; N, 25.38.

*4-Chloro-N-(5-methyl-1H-1,2,4-triazol-3-yl)* benzenesulfonamide (**19**). Yield: 62%. m.p. 219–221 °C. IR (KBr, cm^−1^): 3,468, 3,088; 1,637; 1,384; 1,159; 638. ^1^H-NMR (400 MHz, acetone-d_6_, δ in ppm): 8.04 (d, *J* = 8 Hz, 2H, H2', H6'); 7.75 (d, *J* = 8 Hz, 2H, H3', H5'); 6.71 (s, NH); 2.00 (s, 3H, CH_3_). ^13^C-NMR (100 MHz, DMSO-d_6_, δ in ppm): 161.68; 157.31; 140.27; 135.21; 130.13; 129.35; 13.89. MS *m/z* (%): 272; 111 (100); 75. Anal. Calc. for: C_9_H_9_ClN_4_O_2_S (272.01) C, 39.64; H, 3.33; N, 20.54; found: C, 39.77; H, 3.28; N, 20.56.

*4-Bromo-N-(5-methyl-1H-1,2,4-triazol-3-yl) benzenesulfonamide* (**20**). Yield: 62%. m.p. 170–172 °C. IR (KBr, cm^−1^): 3,468; 3,022; 1,683; 1,392; 1,188. ^1^H-NMR (400 MHz, DMSO-d_6_, δ in ppm): 7.94 (d, *J* = 10 Hz, 2H, H3', H5'); 7.89 (d, *J* = 10 Hz, 2H, H2', H6'); 7.52 (s, NH); 2.00 (s, 3H, CH_3_). ^13^C-NMR (100 MHz, DMSO-d_6_, δ in ppm): 160.18; 156.41; 147.55; 133.17; 130.66; 129.47; 13.56. MS *m/z* (%): 318; 252; 219; 157; 75 (100). Anal. Calc. for: C_9_H_9_BrN_4_O_2_S (351,96) C, 34.08; H, 2.86; N, 17.67; found: C, 34.05; H, 2.66; N, 17.56.

*4-Methyl-N-(5-methyl-1H-1,2,4-triazol-3-yl) benzenesulfonamide* (**21**). Yield: 60%. m.p. 207–209 °C. IR (KBr, cm^−1^): 3,468; 3,089; 1,653; 1,363; 1,159. ^1^H-NMR (400 MHz, DMSO-d_6_, δ in ppm): 7.85 (d, *J* = 8 Hz, 2H, H2', H6'); 7.49 (d, *J* = 8 Hz, 2H, H3', H5'); 7.23 (s, NH); 2.40 (s, 3H, CH_3_); 1.97 (s, 3H, CH_3_). ^13^C-NMR (100 MHz, DMSO-d_6_, δ in ppm): 161.20; 157.20; 146.09; 133.62; 130.32; 127.45; 21.14; 13.89. MS *m/z* (%): 252; 188; 155; 91 (100). Anal. Calc. for: C_10_H_12_N_4_O_2_S (252.06) C, 47.61; H, 4.79; N, 22.21; found: C, 48.01; H, 4.56; N, 22.08.

*N-(5-Methyl-1H-1,2,4-triazol-3-yl) benzenesulfonamide* (**22**). Yield: 59%. m.p. 167–168 °C. IR (KBr, cm^−1^): 3,298; 3,005; 1,687; 1,361; 1,188. ^1^H-NMR (400 MHz, DMSO-d_6_, δ in ppm): 7.96–7.67 (m, 5H, H–Ar); 7.26 (s, NH); 1.98 (s, 3H, CH_3_). ^13^C-NMR (100 MHz, DMSO-d_6_, δ in ppm): 161.31; 157.22; 136.49; 135.12; 129.89; 127.36; 13.86. MS *m/z* (%): 238; 77(100). Anal. Calc. for: C_9_H_10_N_4_O_2_S (238,05) C, 45.37; H, 4.23; N, 23.51; found: C, 45.41; H, 4.03; N, 23.36.

*4-Fluoro-N-(1H-1,2,4-triazol-3-yl)* benzenesulfonamide (**23**). Yield: 51%. m.p. 174–176 °C. IR (KBr, cm^−1^): 3,471; 3,105; 1,649; 1,519; 1,384; 1,139. ^1^H-NMR (400 MHz, DMSO-d_6_, δ in ppm): 8.11-7.54 (m, 4H, H-Ar); 7.62 (s, 1H, H5); 7.40 (s, NH). ^13^C-NMR (100 MHz, DMSO-d_6_, δ in ppm): 165.69 (d, *J* = 254 Hz, C4'); 156.89; 152.71; 132.52; 130.86 (d, *J* = 10 Hz, C2', C6'); 117.73 (d, *J* = 23 Hz, C3', C5'). ^19^F-NMR (376 MHz, DMSO-d_6_, δ in ppm): −101.73 (s, 1F). MS *m/z* (%): 242; 159; 95 (100). Anal. Calc. for: C_8_H_7_FN_4_O_2_S (242,02) C, 39.67; H, 2.91; N, 23.13; found: C, 39.77; H, 2.68; N, 22.95.

*4-Chloro-N-(1H-1,2,4-triazol-3-yl) benzenesulfonamide* (**24**). Yield: 51%. m.p. 200–202 °C. IR (KBr, cm^−1^): NH 3,460; 3,097; 1,643; 1,386; 1,186; 621. ^1^H-NMR (400 MHz, acetone-d_6_, δ in ppm): 8.06 (d, *J* = 8 Hz, 2H, H3', H5'); 7.76 (d, *J* = 8 Hz, 2H, H2', H6'); 7.48 (s, 1H, H5); 6.82 (s, NH). ^13^C-NMR (100 MHz, acetone-d_6_, δ in ppm): 157.00; 152.88; 140.44; 135.02; 130.16; 129.40. MS *m/z* (%): 258; 175; 111 (100). Anal. Calc. for: C_8_H_7_ClN_4_O_2_S (257.99) C, 37.14; H, 2.73; N, 21.66; found: C, 37.35; H, 2.67; N, 21.62.

*4-Bromo-N-(1H-1,2,4-triazol-3-yl) benzenesulfonamide* (**25**). Yield: 55%. m.p. 205–207 °C. IR (KBr, cm^−1^): 3,473; 3,093; 1,614; 1,386; 1,186. ^1^H-NMR (400 MHz, DMSO-d_6_, δ in ppm): 8.31 (s, 1H, H5); 7.99 (s, NH); 7.54 (d, *J* = 10 Hz, 2H, H3', H5'); 7.51 (d, *J* = 10 Hz, 2H, H2', H6'). ^13^C-NMR (100 MHz, DMSO-d_6_, δ in ppm): 150.82; 147.23; 139.24; 130.76; 127.78; 121.90. MS *m/z* (%): 304; 238; 221; 155 (100); 75. Anal. Calc. for: C_8_H_7_BrN_4_O_2_S (301.94) C, 31.70; H, 2.33; N, 18.48; found: C, 29.57; H, 2.88; N, 18.19.

*4-Methyl-N-(1H-1,2,4-triazol-3-yl) benzenesulfonamide* (**26**). Yield: 50%. m.p. 197–199 °C. IR (KBr, cm^−1^): 3,280; 3,035; 1,624; 1,340; 1,151. ^1^H-NMR (400 MHz, DMSO-d_6_, δ in ppm): 7.86 (d, *J* = 8 Hz, 2H, H2', H6'); 7.57 (s, 1H, H5); 7.49 (d, *J* = 8 Hz, 2H, H3', H5'); 7.32 (s, NH); 2.40 (s, 3H, CH_3_). ^13^C-NMR (100 MHz, DMSO-d_6_, δ in ppm): 156.88; 152.47; 146.30; 133.42; 130.35; 127.48; 21.14. MS *m/z* (%): 223; 149 (100). Anal. Calc. for: C_9_H_10_N_4_O_2_S (238,05) C, 45.37; H, 4.23; N, 23.51; found: C, 45.40; H, 4.01; N, 23.53.

*N-(1H-1,2,4-Triazol-3-yl) benzenesulfonamide* (**27**). Yield: 57%. m.p. 163–165 °C. IR (KBr, cm^−1^): 3,278; 3,155; 2,775; 1,693; 1,369; 1,186. ^1^H-NMR (400 MHz, DMSO-d_6_, δ in ppm): 8.32 (s, 1H, H5); 8.05 (s, NH); 7.62–7.31 (m, 5H, H–Ar). ^13^C-NMR (100 MHz, DMSO-d_6_, δ in ppm): 150.79; 147.89; 139.21; 128.63; 127.74; 125.46. MS *m/z* (%): 223; 149 (100). Anal. Calc. for: C_8_H_8_N_4_O_2_S (224.03) C, 42.85; H, 3.60; N, 24.99; found: C, 41.51; H, 4.77; N, 21.96.

## 4. Conclusions

In this work, we designed and synthesized a set of 15 compounds analogs to sulfadoxine, sulfadiazine, and sulfalene in order to identify their antiparasitic profiles, which could then be correlated with their molecular properties. The 3-amine-1*H*-1,2,4-triazoles **2–4** were prepared in good yield (91%–99%) from commercially available raw materials. The 1*H*-1,2,4-triazol-3-yl benzenesulfonamide derivatives **13–27** were synthesized using compounds **2–4**, which were already prepared on a large scale by our research group. This simple methodology afforded the sulfonamide derivatives using the appropriate sulfonyl chlorides. According to the docking calculations, compounds with CF_3_ groups at position 5 of the triazole ring must be carefully considered when designing new 1*H*-1,2,4-triazol-3-yl benzenesulfonamide derivatives as potential inhibitors of *Pf*DHPS. Moreover, halogen groups at the 4' position increase the ligand-enzyme interaction. Overall, these results suggest that *N*-(5-trifluoromethyl)-1*H*-1,2,4-triazol-3-yl benzenesulfonamides are potential lead compounds for the development of new antimalarial drugs. The results reported herein should be useful in guiding future efforts to discover compounds with increased antimalarial activity. Consequently, the reported compounds are currently undergoing *in vitro* antimalarial tests.
